# METTL3‐m^6^ A methylase regulates the osteogenic potential of bone marrow mesenchymal stem cells in osteoporotic rats via the Wnt signalling pathway

**DOI:** 10.1111/cpr.13234

**Published:** 2022-04-25

**Authors:** Tianli Wu, Hui Tang, Jianghua Yang, Zhihao Yao, Long Bai, Yuping Xie, Qing Li, Jingang Xiao

**Affiliations:** ^1^ Department of Oral Implantology The Affiliated Stomatological Hospital of Southwest Medical University Luzhou China; ^2^ Department of Oral and Maxillofacial Surgery The Affiliated Hospital of Southwest Medical University Luzhou China; ^3^ Luzhou Key Laboratory of Oral & Maxillofacial Reconstruction and Regeneration The Affiliated Stomatological Hospital of Southwest Medical University Luzhou China; ^4^ Department of Medical Technology, Faculty of Associated Medical Sciences Chiang Mai University Chiang Mai Thailand

## Abstract

**Objectives:**

Bone marrow mesenchymal stem cells (BMSCs) hold a high osteogenic differentiation potential, but the mechanisms that control the osteogenic ability of BMSCs from osteoporosis (OP‐BMSCs) need further research. The purpose of this experiment is to discuss the osteogenic effect of *Mettl3* on OP‐BMSCs and explore new therapeutic target that can enhance the bone formation ability of OP‐BMSCs.

**Materials and Methods:**

The bilateral ovariectomy (OVX) method was used to establish the SD rat OP model. Dot blots were used to reveal the different methylation levels of BMSCs and OP‐BMSCs. Lentiviral‐mediated overexpression of *Mettl3* was applied in OP‐BMSCs. QPCR and WB detected the molecular changes of osteogenic‐related factors and Wnt signalling pathway *in vitro* experiment. The staining of calcium nodules and alkaline phosphatase detected the osteogenic ability of OP‐BMSCs. Micro‐CT and histological examination evaluated the osteogenesis of *Mettl3* in OP rats *in vivo*.

**Results:**

The OP rat model was successfully established by OVX. Methylation levels and osteogenic potential of OP‐BMSCs were decreased in OP‐BMSCs. *In vitro* experiment, overexpression of *Mettl3* could upregulate the osteogenic‐related factors and activate the Wnt signalling pathway in OP‐BMSCs. However, osteogenesis of OP‐BMSCs was weakened by treatment with the canonical Wnt inhibitor Dickkopf‐1. Micro‐CT showed that the *Mettl3*(+) group had an increased amount of new bone formation at 8 weeks. Moreover, the results of histological staining were the same as the micro‐CT results.

**Conclusions:**

Taken together, the methylation levels and osteogenic potential of OP‐BMSCs were decreased in OP‐BMSCs. *In vitro* and *in vivo* studies, overexpression of *Mettl3* could partially rescue the decreased bone formation ability of OP‐BMSCs by the canonical Wnt signalling pathway. Therefore, *Mettl3* may be a key targeted gene for bone generation and therapy of bone defects in OP patients.

## Graphical abstract

In this study, the osteoporosis rat model was successfully established by OVX. OP‐BMSCs were successfully isolated and cultured from the femur of OP rat. Lentiviral‐mediated overexpression of *Mettl3* could partially rescue the impaired osteogenic ability of OP‐BMSCs by activating the canonical Wnt signalling pathway *in vitro* and *in vivo* *.*

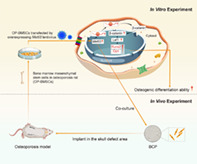

## 1 INTRODUCTION

As the worldwide population is rapidly aging, osteoporosis (OP) could become a global health‐care challenge.^1^, ^2^ OP has low bone mineral density and structural degradation of bones and increases the risk of fracture in OP patients.^1^, ^3^, ^4^, ^5^ Postmenopausal women suffer from OP due to the rapid decline in oestrogen levels.^6^, ^7^ At the same time, the bone loss caused by menopause usually precedes age‐related bone loss, so postmenopausal women may develop osteoporotic fractures earlier than men of the same age.^1^, ^8^, ^9^, ^10^ Treatments for osteoporotic fractures include drug, non‐drug and stem cell therapies.^11^, ^12^, ^13^ However, medical treatment has the disadvantages of not reversing the existing bone loss and potentially causing serious side effects.^14^ Therefore, stem cell therapy is a research hotspot amongst treatments for OP‐related bone defects.^15^, ^16^

Bone marrow mesenchymal stem cells (BMSCs) are one of the most effective mesenchymal stem cell types for treating OP.^17^ Pino et al. found enhanced adipogenic differentiation and weakened osteogenic differentiation of BMSCs in postmenopausal OP patients (OP‐BMSCs),^18^ resulting in an imbalance between osteogenic and adipogenic differentiation, which disrupts the activity and microenvironment of OP‐BMSCs.^19^ Therefore, it is essential to find ways to reactivate the bone formation of OP‐BMSCs and inactivate the formation of adipocytes. This issue is a key and difficult aspect of treating osteoporotic fractures with autologous OP‐BMSCs transplantation.

Since its discovery in the 1970s, N6‐methyl‐adenosine (m^6^A) has been recognized as the most common internal chemical modification of mRNAs in eukaryotes. The m^6^A methyltransferase complex includes Wilms tumour 1‐associating protein (WTAP), methyltransferase‐like 3 (METTL3), methyltransferase‐like 14 (METTL14) and m^6^A demethylase, including fat mass and obesity‐associated protein (FTO) and ALKB homologue 5 (ALKBH5).^20^, ^21^ M^6^A is dynamically regulated by methylases and demethylases and controls cellular processes, and the translation of mRNAs involved in cell metabolism, cell growth and disease development.^22^ It is well known that the m^6^A modification has multiple biological functions. Furthermore, METTL3 is an important m^6^A methylase that can catalyse the conversion of adenosine to m^6^A. METTL3 is a 70‐kDa protein containing methylation‐active catalytic residues.^23^ The expression ratios of the components of the m^6^A methyltransferase complex vary greatly amongst tissues or cell types, indicating that they have different biological functions and methylation activities. Studies have shown that METTL3 deficiency decreases m^6^A levels, attenuating the normal lineage and disrupting cell cycle progression.^24^, ^25^ However, the role of *Mettl3* in bone homeostasis is little known.

The Wnt signalling pathway is a pivotal regulator of bone differentiation, development and homeostasis of BMSCs.^26^, ^27^, ^28^ Todd et al. found that Wnt16 was significantly reduced in the BMSCs of ovariectomized (OVX) mice.^29^ Wnt signalling pathway activation can enhance Wnt‐related genes, restore the osteogenic potential of BMSCs and attenuate bone loss.^30^ Similarly, Dickkopf‐1 (DKK1) can reduce the osteogenic potential of BMSCs and cause bone loss.^31^, ^32^ Previous studies have shown that *Mettl3* could regulate the Wnt/β‐catenin pathway.^33^, ^34^ However, the regulatory mechanism of *Mettl3* influences the bone differentiation of OP‐BMSCs through the canonical Wnt pathway is unknown.

The experiment was to establish an OP model in Sprague Dawley (SD) rats with the OVX method, detect *Mettl3* expression in OP‐BMSCs from the OP model and control SD rats at molecular levels and then assess the effects of lentiviral‐mediated *Mettl3* overexpression. Finally, the Wnt signalling pathway was blocked by DKK1. Changes of the runt‐related transcription factor 2 (Runx2) and osteopontin (Opn); the canonical Wnt target genes β‐Catenin (β‐Catenin), lymphoid enhancing factor 1 (Lef1) and glycogen synthase kinase‐3β (Gsk‐3β); and the phosphorylation of glycogen synthase kinase‐3β (P‐Gsk‐3β) were observed.

We investigated the mechanism of *Mettl3* on the osteogenesis of OP‐BMSCs on the transcriptional level by overexpressing *Mettl3* and explored further methods of enhancing the osteogenic capacity of OP‐BMSCs. Moreover, we examined the potential of *Mettl3* to be a therapeutic target for OP‐related bone defects.

## 2 MATERIAL AND METHODS

### 2.1 The OP animal model

Thirty four‐week‐old SD female rats were purchased from Southwest Medical University (Luzhou, China). About 1% of sodium pentobarbital (30 mg/kg) was used to anaesthetize the rats, and OVX was performed to establish the OP SD rat model. Southwest Medical University Ethical Committee (20180391222) reviewed and approved experiment. The experiment was conducted under the guideline of the Care and Use of Laboratory Animals (Ministry of Science and Technology of China, 2006).

### 2.2 Micro‐computed tomography analysis, Haematoxylin and eosin staining and Masson staining

Femurs were dissected from the OVX and control rats 3 months after OVX. The entire femur was scanned using an instrument (SCANCO Medical). Then, femurs samples were stained by haematoxylin and eosin (H&E) and Masson reagents after the decalcification was completed.

### 2.3 Isolation and culture of BMSCs and OP‐BMSCs

A sterile syringe filled in culture medium contained 10% foetal bovine serum (FBS; Schaumburg) was used to repeatedly flush the bone marrow cavity of the femur until the bone marrow cavity turned white and no bone marrow tissue remained. Finally, the culture medium containing bone marrow tissue was cultured in 25 cm^2^ culture flasks. The liquid was 50% changed on the first 3 days and then completely changed on day 4. The cells were passaged to third passage for experiments.

### 2.4 Characterization of isolated OP‐BMSCs by flow cytometry

Third passage OP‐BMSC suspension was resuspended in phosphate‐buffered saline (PBS). One group was incubated with fluorophore‐conjugated anti‐CD29, ‐CD90, ‐CD44, ‐CD31, ‐CD45 and ‐CD34 antibodies, and another group was used as controls without fluorophore antibodies. We used FITC anti‐rat CD29, and CD44, APC anti‐rat CD45, PE anti‐rat CD31, and CD34, and PECY7 anti‐rat CD90 antibodies (BioLegend) to conjugate with the CD29, CD44, CD45, CD31, CD34 and CD90 of cells. Fluorescence‐activated cell sorter (FACS Calibur) was used to detect cells after incubating in the dark for 30 min.

### 2.5 Alkaline phosphatase and alizarin red staining

Third passage BMSCs were cultured in a 12‐well plate, and osteogenic induction fluid (10% FBS, 1% β‐glycerophosphate, 1% penicillin–streptomycin, 1% glutamine, 0.2% ascorbate and 0.01% dexamethasone, Cyagen) was added when the cell confluence reached 80%. After 3 and 5 days of osteogenic induction, alkaline phosphatase (ALP) activity was detected with the BCIP/NBT Alkaline Phosphatase Kit (Shenggong, Shanghai, China). The calcium nodules were stained by Alizarin Red Staining (Cyagen) on the 21st day of osteogenic induction.

### 2.6 Dot blot of m^6^ A RNA modification levels

Briefly, total RNA was collected by TRIzol reagent (Ambion), then a Dynabeads® mRNA Purification Kit (Thermo Fisher Scientific) was used to extract mRNA, which was heat extracted at 95°C for 3 min. Then, 2 μl of mRNA was directly dropped onto a Hybond‐N+ (Sigma‐Aldrich) membrane optimized for nucleic acid transfer. The spotted mRNA was crosslinked to the membrane using a Stratalinker 2400 UV Crosslinker in the autocrosslink mode (1200 μJ [×100]; 25–50 s). PBS was used to rinse the membrane for 5 min to wash away unbound mRNA. Anti‐m^6^A antibody (Synaptic Systems) was incubated with the membrane overnight at 4°C after blocking the bands. Next, the membrane was incubated with goat anti‐rabbit IgG‐HRP (Signalway Antibody) for 1 h, then submerged in chemiluminescence liquid, and an ECL chemiluminescence detection system (iBrightCL1000, Singapore) was used to obtain images.

### 2.7 Immunofluorescence staining

Third‐passage BMSCs were incubated in a 20‐cm glass bottom cell culture dish. After 3 days of osteogenic induction, cells were fixed, washed, blocked and incubated in anti‐RUNX2 and anti‐OPN (both 1:100, Abcam) for 12 h. The rest of the experimental procedures were performed in a dark environment. Next day, the goat antibody (1:200, Beyotime) was incubated with the cells for 1 h. The cytoskeleton was stained with phalloidin (1:100, cytoskeleton, America) for 30 min. The nuclei were stained by 4′6‐diamidino‐2‐phenylindole (DAPI, Beyotime, China) for 15 min, and the images were captured by microscope (Leica).

### 2.8 Determination of the MOI of the *Mettl3* overexpression lentivirus

Heyuan Biotechnology Co., Ltd completed the construction of *Mettl3* lentivirus vectors. For *Mettl3* overexpression, the *Mettl3* sequence ((forward) 5′‐CGCAAATGGGCGGTAGGCGTG‐3′ and (reverse) 5′‐AAGAACGGAGCCGGTTGGCG‐3′) was amplified and subcloned into the GL119 pSLenti‐CMV‐MCS‐3xFLAG‐PGK‐Puro‐WPRE lentiviral vector to get pSLenti‐CMV‐Mettl3‐3xFLAG‐PGK‐Puro‐WPRE lentivirus. There were CON group (no lentivirus), *Mettl3*(−) group (lentivirus without *Mettl3* sequence) and *Mettl3*(+) group (lentivirus with *Mettl3* sequence) according to whether transfected with *Mettl3* lentivirus. The MOI value was 0, 10, 20, 40, 60, 80 and 100. A fluorescence microscope was used to observe and record the cell morphologies and fluorescence intensities to determine the optimal MOI of the lentivirus after transduction for 72 h.

### 2.9 Real‐time quantitative polymerase chain reaction

First, RZ lysate (Tiangen) was used to get RNA, and then the RevertAid First Strand cDNA Synthesis Kit (Thermo) turned total RNA into cDNA. Shenggong Bioengineering. Co., Ltd synthesized the gene primers and the sequences are shown in Table 1. All quantitative polymerase chain reaction (qPCR) reactions were carried out in 96‐well plates according to PrimeScript RT‐PCR Kit (Takara Bio).

**TABLE 1:** The specific gene primer sequences

Genes	Primers	Sequence (5′ → 3′)
*Gapdh*	Forward	TTTGAGGGTGCAGCGAACTT
	Reverse	ACAGCAACAGGGTGGTGGAC
*Mettl3*	Forward	CTCAGATCTCGCCTTAACCTTGCC
	Reverse	GACAGCTTGGAGTGGTCAGCATAG
*Wtap*	Forward	AGAACATCCTTGTCATGCGGCTAG
	Reverse	CGGCTTCAAGCTGTGCAATACG
*Fto*	Forward	AATGAAGACGCTGTGCCGTT
	Reverse	GAAGCTGGACTCGTCATCGC
*Mettl14*	Forward	AGTTTGGGAGCTGAGAGTG
	Reverse	GTATCATAGGAAGCCCTGCA
*Ythdf1*	Forward	TTGGTGGCACAGTTGTTGAT
	Reverse	ACCATTGCCAGAAAGGACAC
*Opn*	Forward	CACTCCAATCGTCCCTACA
	Reverse	CTTAGACTCACCGCTCTTCAT
*Lef‐1*	Forward	CAGACCTGTCACCCTTCAGC
	Reverse	GTGAGACGGATTGCCAAACG
*Runx2*	Forward	GAACCAAGAAGGCACAGAC
	Reverse	AATGCGCCCTAAATCACTG
*β‐Catenin*	Forward	ACTCTAGTGCAGCTTCTGGGTTCTG
	Reverse	CTCGGTAATGTCCTCCCTGTCA

### 2.10 Western blot assay

Total protein extraction of BMSCs and OP‐BMSCs was processed by the Protein Extraction Kit (Keygen Biotech). The proteins were migrated to polyvinylidene difluoride (PVDF) membranes and combined with specific antibodies to GAPDH (ab181602), OPN (ab8448), RUNX2 (ab92336), β‐CATENIN (ab32572), METTL3 (ab195352) (all Abcam), LEF1 (C12A5) GSK (9832S) and P‐GSK (9323S) (Cell Signalling Technology). Then, goat anti‐rabbit or anti‐mouse antibodies (Signalway Antibody) were combined with the specific antibodies for 1 h. An Affinity ECL western blotting substrate (Affinity) was used to develop the immunoreactive bands and visualized by the ECL system.

### 2.11 Cell Counting Assay and scanning electron microscopy

The proliferation of OP‐BMSCs seeded on BCP was detected by cell counting kit‐8 (CCK‐8, APExBIO) according to the supplied protocols after co‐cultured for days. Then, the BCP samples were observed by SEM after dehydration.

### 2.12 BCP seeded with OP‐BMSCs transplanted into a critical‐sized calvarial defect OP rat model

OP‐BMSCs transduced with *Mettl3* overexpression lentivirus, untransduced with lentivirus and transduced with *Mettl3* overexpression control lentivirus were cultured. Then, a 2‐ml cell suspension (5 × 10^5^ cells/ml) was cultured with BCP in a 12‐well plate and incubated for 3 days. OP rats were fixed prone, the skin was prepared and the skull was disinfected, resulting in a full‐thickness defect of 8 mm. BCP incubated with OP‐BMSCs was implanted in the skull defect area. After 8 weeks, skull specimens were taken after the rats were euthanized.

### 2.13 Statistical Analysis

The representative data are provided as mean ± standard deviation (SD). All statistical analyses were performed using SPSS 19.0 software (IBM). It is statistically significant at *p* < 0.05.

## 3 RESULTS

### 3.1 The OP rats were successfully constructed

**FIGURE 1:**
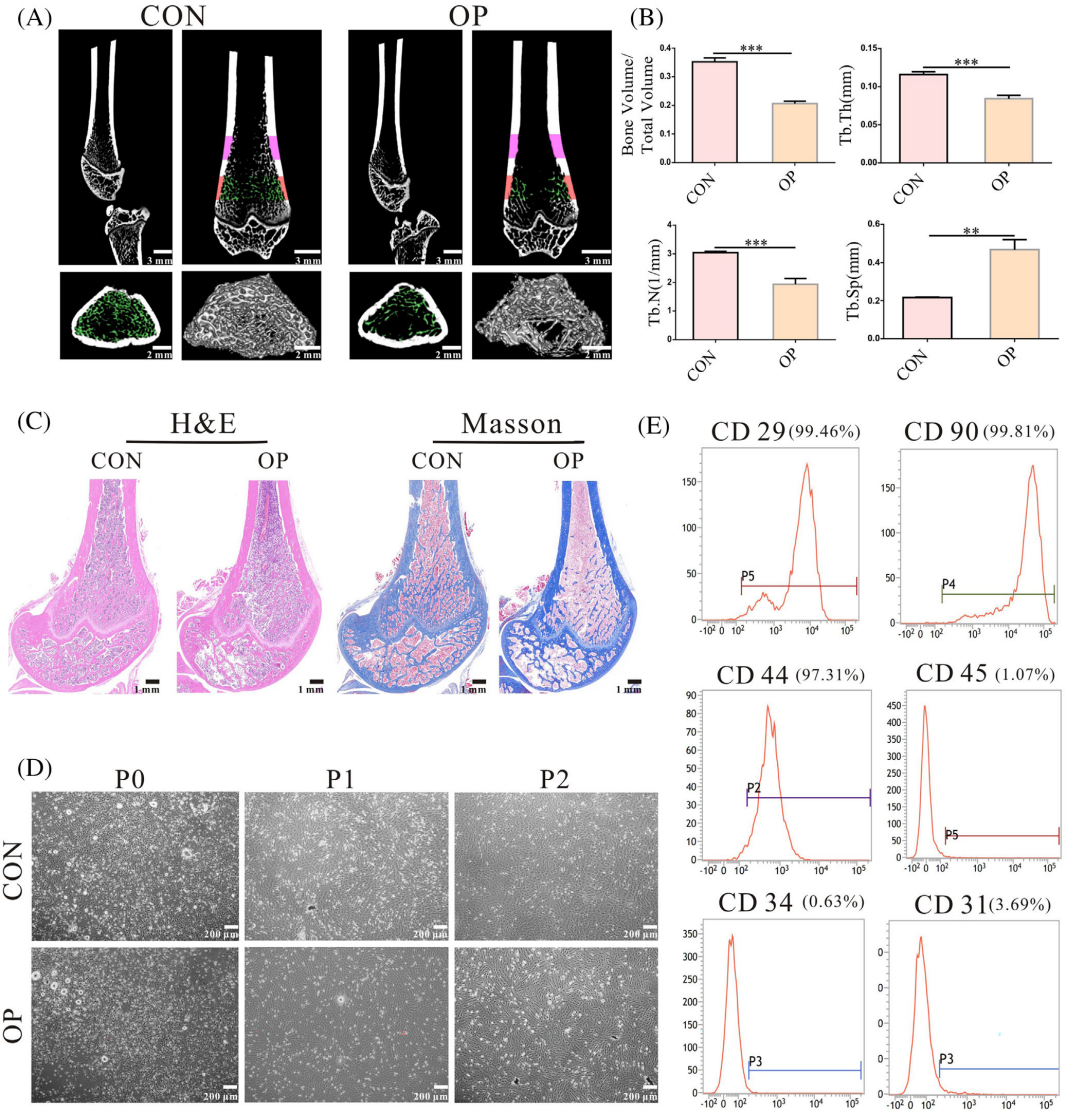
The OP rats were successfully constructed and the successful isolation and culture of BMSCs and OP‐BMSCs. (A, B) Micro‐CT analysis of the femur showed that OVX femoral trabecular number was reduced and a trabecular fracture was observed. (C) H&E staining and Masson staining showed that the OVX femoral trabecular number had decreased, bone thinning, areas of trabecular bone fracture, perforations, loss of the normal arch‐like structure, altered continuity and expanded bone marrow cavity. (D) Primary culture and subculture of OP‐BMSCs and BMSCs. The cell morphology was uniformly long spindle‐like. (E) The expression of the surface markers CD 29, CD 90, CD 44, CD 31, CD 45 and CD 34 in OP‐BMSCs were shown by FACS. Data represent the mean ± SD (*n* ≥ 3). (*p < 0.05,**p < 0.01, ***p < 0.001)

Twelve weeks after OVX, 3‐D renderings of high‐resolution Micro‐computed tomography (micro‐CT) scans of the proximal femur showed that compared with sham‐operated rats, the trabecular bone of the femur of OVX rats was broken, the number was reduced, the arrangement was sparse and irregular and the bone marrow cavity was enlarged (Figure 1A). The results of the morphological analysis show a decrease in trabecular thickness (Tb.Th), bone tissue volume/tissue volume (BV/TV), and trabecular number (Tb.N) and an increase in trabecular separation (Tb.Sp) in OVX rats (Figure 1B). H&E staining was used to analyse the trabecular bone morphology. The trabecular thickness of the control group was uniformly increased, and trabecular fractures were rarely observed. In comparison, Masson staining showed that femurs of OVX rats were characterized by thinning of the trabecular bone fracture area, perforation, loss of normal arcuate structure, and changes in the continuity and enlargement of the bone marrow cavity, all of which indicated bone resorption and defects in bone formation (Figure 1C). These results indicated the successful establishment of the OP rat model.

### 3.2 Successful isolation and culture of BMSCs and OP‐BMSCs

We obtained femurs of sham‐operated and OP rats to culture BMSCs and OP‐BMSCs, respectively. The primary generation of BMSCs and OP‐BMSCs was passaged to the P3 generations. The cells were uniformly long spindle‐like (Figure 1D). Third‐generation OP‐BMSCs were analysed by flow cytometry. The haematopoietic lineage markers CD 34, CD 45 and endothelial cell marker CD 31 of OP‐BMSCs were approximately 95% negative, and the mesenchymal markers CD 29, CD 90 and CD 44 were approximately 95% positive (Figure 1E). These results confirmed that OP‐BMSCs had self‐renewal capacity and were of mesenchymal lineages.

### 3.3 Decreased osteogenic differentiation potential in OP‐BMSCs

**FIGURE 2:**
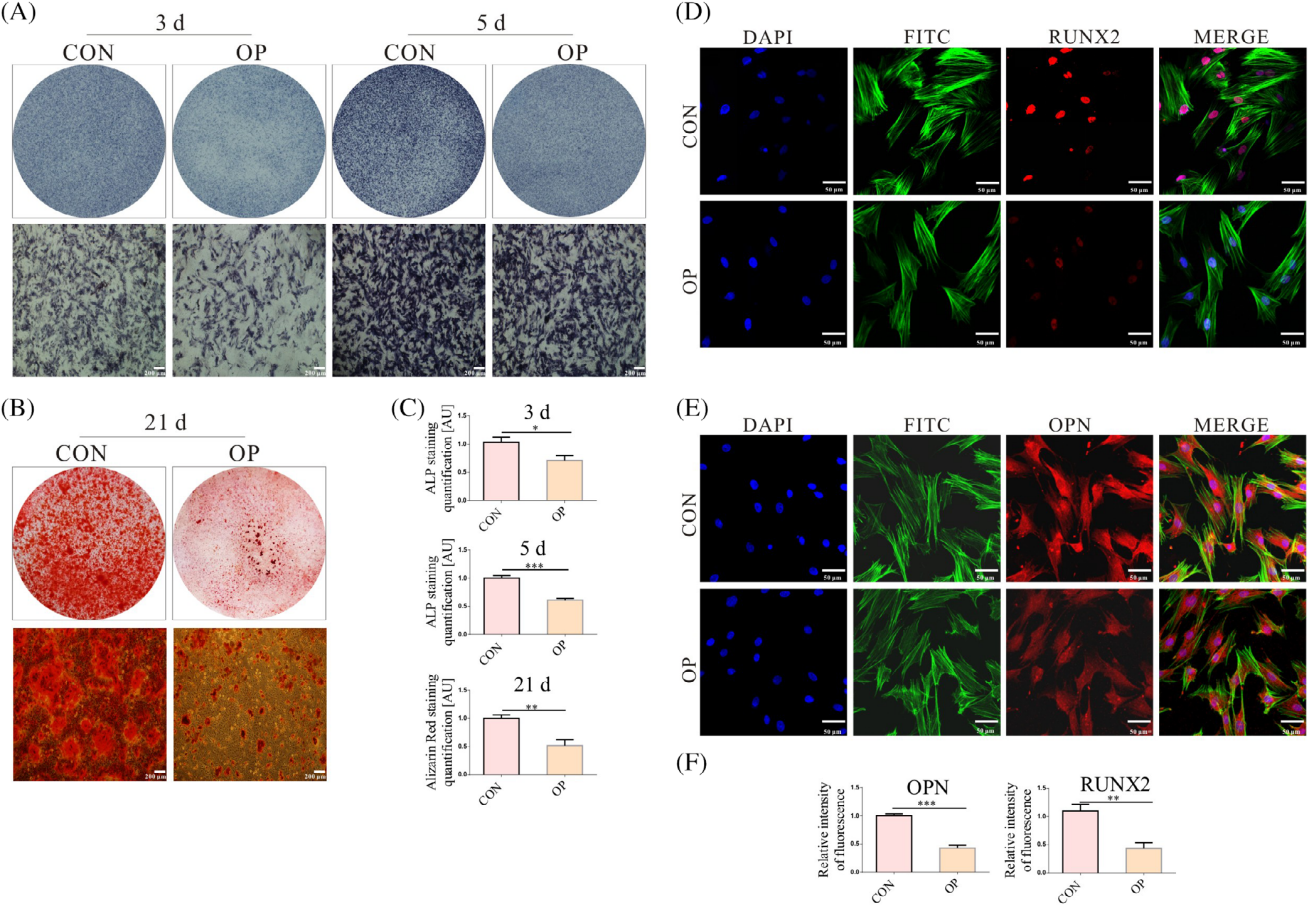
The osteogenic differentiation potential was decreased in OP‐BMSCs. (A) ALP staining showed less alkaline phosphatase activity in OP‐BMSCs after osteogenic induction for 3 and 5 days. (B) Alizarin red staining showed fewer mineralized nodules after osteogenic induction for 21 days. (C) Semi‐quantitative analysis of ALP staining, Alizarin red staining by utilizing ImageJ. (D) Immunofluorescence staining for RUNX2 after osteogenic induction for 3 days. (E) Immunofluorescence staining for OPN after osteogenic induction for 3 days. (F) Semi‐quantitative analysis of immunofluorescence staining by utilizing ImageJ. Data represent the mean ± SD (*n* ≥ 3). (*p < 0.05,**p < 0.01, ***p < 0.001)

We next assessed the bone formation differences of BMSCs and OP‐BMSCs by culturing them in osteogenic induction medium. After induction, ALP staining showed reduced ALP activity in OP‐BMSCs. (Figure 2A,C). Alizarin red staining showed that calcium nodules had formed in both cell types, but OP‐BMSCs had fewer calcium nodules than BMSCs did (Figure 2B,C). These results tested that OP‐BMSCs had impaired osteogenic ability. Next, we used immunofluorescence staining to detect the level of RUNX2 and OPN in OP‐BMSCs and BMSCs. These results showed decreased nuclear RUNX2 expression in OP‐BMSCs compared with BMSCs (Figure 2D,F). Moreover, there was reduced cytoplasmic OPN staining in OP‐BMSCs compared with BMSCs (Figure 2E,F).

### 3.4 Downregulation of m^6^ A methylation level and Wnt signalling pathway in OP‐BMSCs

**FIGURE 3:**
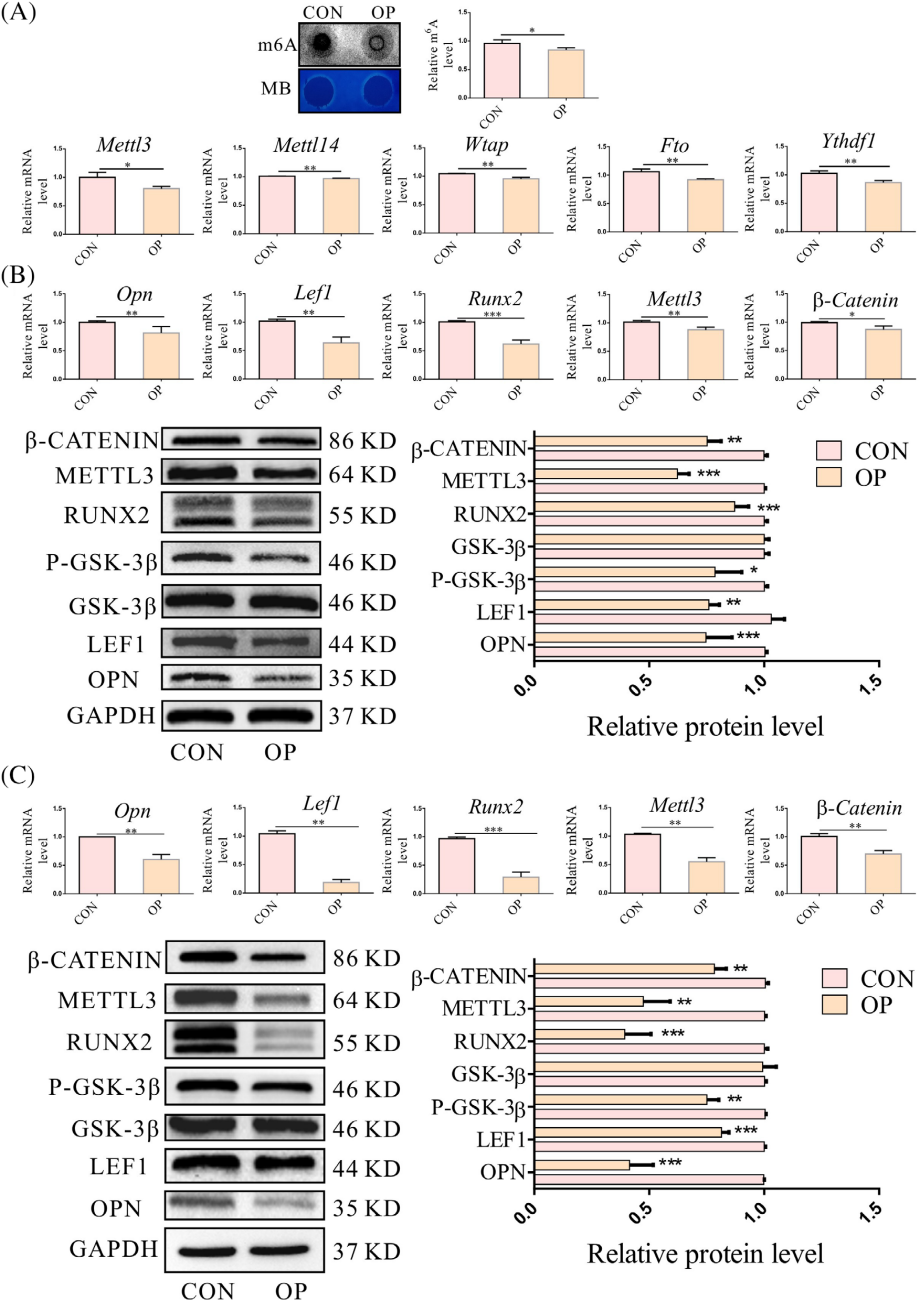
Expression of m^6^A methylation level and Wnt signalling pathway were downregulated in OP‐BMSCs. (A) The mRNA methylation level of OP‐BMSCs was lower than that of BMSCs, methylene blue staining (MB) was used as a loading control. The gene expression of *Mettl3*, *Mettl14*, *Wtap*, *Fto* and *Ythdf1* was lower in OP‐BMSCs compared to BMSCs. (B) Gene and protein expression of Mettl3, Runx2, Opn, Lef1, P‐Gsk‐3β and β‐Catenin were decreased after osteogenic induction for 3 days in OP‐BMSCs. (C) Gene and protein expression of Mettl3, Runx2, Opn, Lef1, P‐Gsk‐3β and β‐Catenin were also decreased after osteogenic induction for 5 days in OP‐BMSCs. Data represent the mean ± SD (*n* ≥ 3). (*p < 0.05,**p < 0.01, ***p < 0.001)

We detected m^6^A methylation in OP‐BMSCs and BMSCs using an m^6^A mRNA dot blot and by measuring the RNA expression of related m^6^A RNA methylation factors. The dot blot results showed reduced levels of m^6^A methylated mRNA in OP‐BMSCs. The RNA expression levels of *Mettl3*, *Mettl14* and Wtap in OP‐BMSCs were all decreased, levels of the demethylase *Fto* were decreased and expression of reader *Ythdf1* was decreased (Figure 3A). Together, these data showed reduced levels of m^6^A methylation in OP‐BMSCs compared with BMSCs and significantly decreased *Mettl3* expression in OP‐BMSCs. Next, we detected the expression of *Mettl3*, osteogenic‐related genes and Wnt target genes in OP‐BMSCs after culture in osteogenic differentiation media for 3 and 5 days. Meanwhile, the protein and mRNA level of Mettl3, Opn, Runx2, β‐Catenin, Lef1, and P‐Gsk‐3β were decreased in OP‐BMSCs compared with BMSCs after culture in osteogenic induction medium for 3 days (Figure 3B). The molecular changes after 5 days of osteogenic induction were consistent with the changes after 3 days (Figure 3C). Thus, the overall levels of m^6^A during the osteogenic differentiation of OP‐BMSCs were decreased along with the downregulation of the methylating enzyme METTL3.

### 3.5 Overexpression of *Mettl3* improved the osteogenic ability of OP‐BMSCs by activating Wnt signalling pathway *in vitro*

**FIGURE 4:**
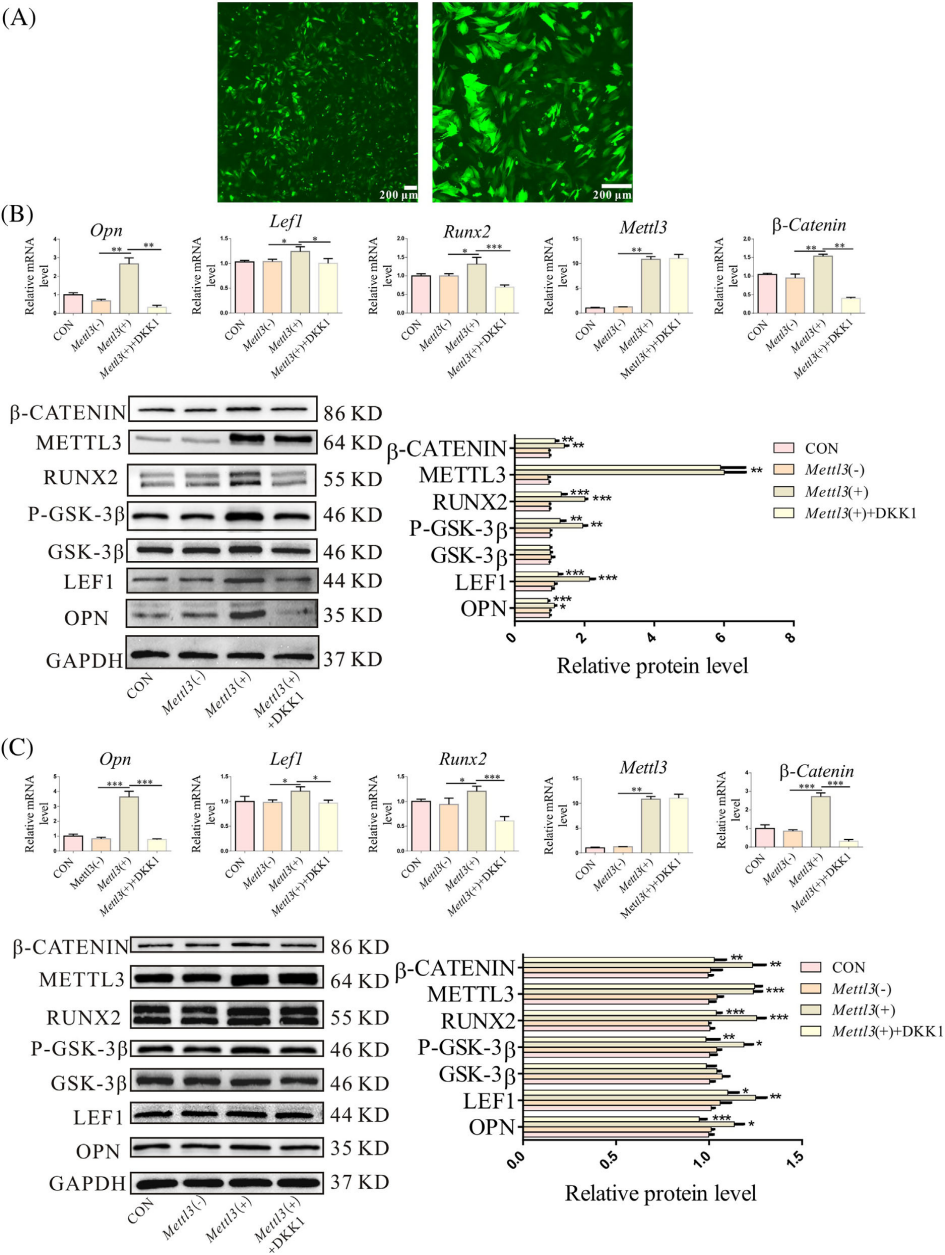
Overexpression of *Mettl3* improved the osteogenic ability of OP‐BMSCs by activating the canonical Wnt signalling pathway *in vitro*. (A) *Mettl3* overexpression lentivirus transfected into OP‐BMSCs for 72 h with an MOI value of 60. (B) Relative gene and protein expression of Mettl3, Runx2, Opn, Lef1, P‐Gsk‐3β and β‐Catenin were increased after *Mettl3* overexpressing in OP‐BMSCs for osteogenic induction of 3 days. (C) After *Mettl3* was overexpressed in OP‐BMSCs, the relative gene and protein expression of Mettl3, Runx2, Opn, Lef1, P‐Gsk‐3β and β‐Catenin were increased after osteogenic induction for 5 days. Data represent the mean ± SD (*n* ≥ 3). (*p < 0.05,**p < 0.01, ***p < 0.001)

To determine whether METTL3‐mediated regulation of osteogenic differentiation in OP‐BMSCs was dependent on the m^6^A modification, a *Mettl3* overexpression lentivirus was generated by Heyuan Biotechnology Co., Ltd (Shanghai, China). The fluorescence signal was strongest in the *Mettl3* (+) groups after transduction for 72 h at a MOI value of 60. Thus, the *Mettl3* overexpression lentivirus was used for subsequent experiments at a MOI of 60 (Figure 4A). Next, qPCR and WB were used to confirm that *Mettl3* had good overexpression efficiency. The expression of *Mettl3* in *Mettl3* lentivirus‐transduced OP‐BMSCs was upregulated. Additionally, we used DKK1 to detect whether the osteogenic differentiation capacity of BMSCs is related to Wnt signalling pathway. The results showed that DKK1 did not change *Mettl3* expression in the *Mettl3* overexpression group (Figure 4B).

Next, we detected the bone‐related regulatory effect of *Mettl3* of OP‐BMSCs. After transduction with the *Mettl3* overexpression lentivirus for 3 days, Opn and Runx2 were enhanced, the weakened osteogenic ability of OP‐BMSCs was rescued and P‐Gsk‐3β, Lef1 and β‐Catenin were increased, suggesting activation of Wnt signalling pathway. In contrast, DKK1 inhibited the canonical Wnt signalling pathway, as evidenced by decreased levels of P‐Gsk‐3β, Lef1, β‐Catenin, Opn and Runx2. Therefore, there was also decreased osteogenic ability (Figure 4B). Similar results were found after osteogenic induction for 5 days (Figure 4C).

**FIGURE 5:**
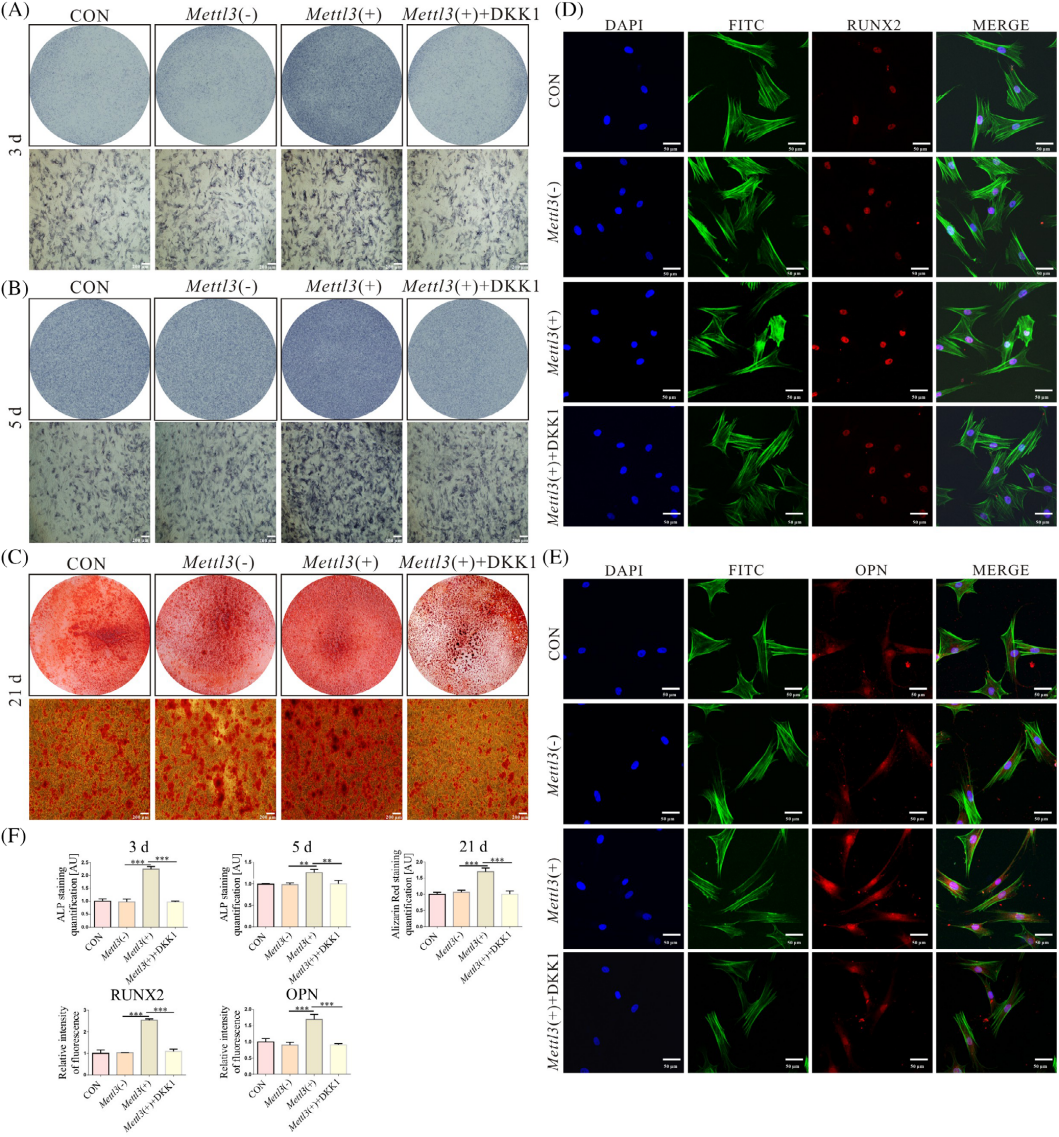
The upregulated osteogenesis of OP‐BMSCs after overexpression of *Mettl3* in vitro. (A, B) ALP staining showed that alkaline phosphatase activity of the *Mettl3*(+) group was higher than that of CON, *Mettl3*(−) and *Mettl3*(+) + DKK1 groups after osteogenic induction for 3 and 5 days. (C) Alizarin red staining showed that the bone differentiation potential of the *Mettl3*(+) group was increased compared to that of CON, *Mettl3*(−) and *Mettl3*(+) + DKK1 groups after osteogenic induction for 21 days. (D) Immunofluorescence staining for RUNX2 after osteogenic induction for 3 days. (E) Immunofluorescence staining for OPN after osteogenic induction for 3 days. (F) Semi‐quantitative analysis of ALP staining, Alizarin red staining and immunofluorescence staining by utilizing ImageJ. Data represent the mean ± SD (*n* ≥ 3). (*p < 0.05,**p < 0.01, ***p < 0.001)

Subsequently, we used ALP, Alizarin red and immunofluorescence staining to detect the role of *Mettl3*‐overexpressing. After osteogenesis induction for 3 days, ALP staining showed increased ALP activity in the *Mettl3*(+) group and weakened ALP activity in the *Mettl3*(+) + DKK1 group (Figure 5A,F). After osteogenesis induction for 5 days, there was more intense ALP staining than after 3 days (Figure 5B,F). Alizarin red staining showed increased numbers of calcium nodules in the *Mettl3*(+) group, whilst calcium nodules were chronically reduced in the *Mettl3*(+) + DKK1 group (Figure 5C,F). Moreover, the protein expression of OPN and RUNX2 increased in the *Mettl3*(+) group, whilst both were decreased in the *Mettl3*(+) + DKK1 group (Figure 5D–F).

In summary, the methylase METTL3 had a positive bone‐related regulatory effect of OP‐BMSCs through the canonical Wnt signalling pathway.

### 3.6 Overexpression of *Mettl3* upregulated the osteogenic ability of OP‐BMSCs *in vivo*

**FIGURE 6:**
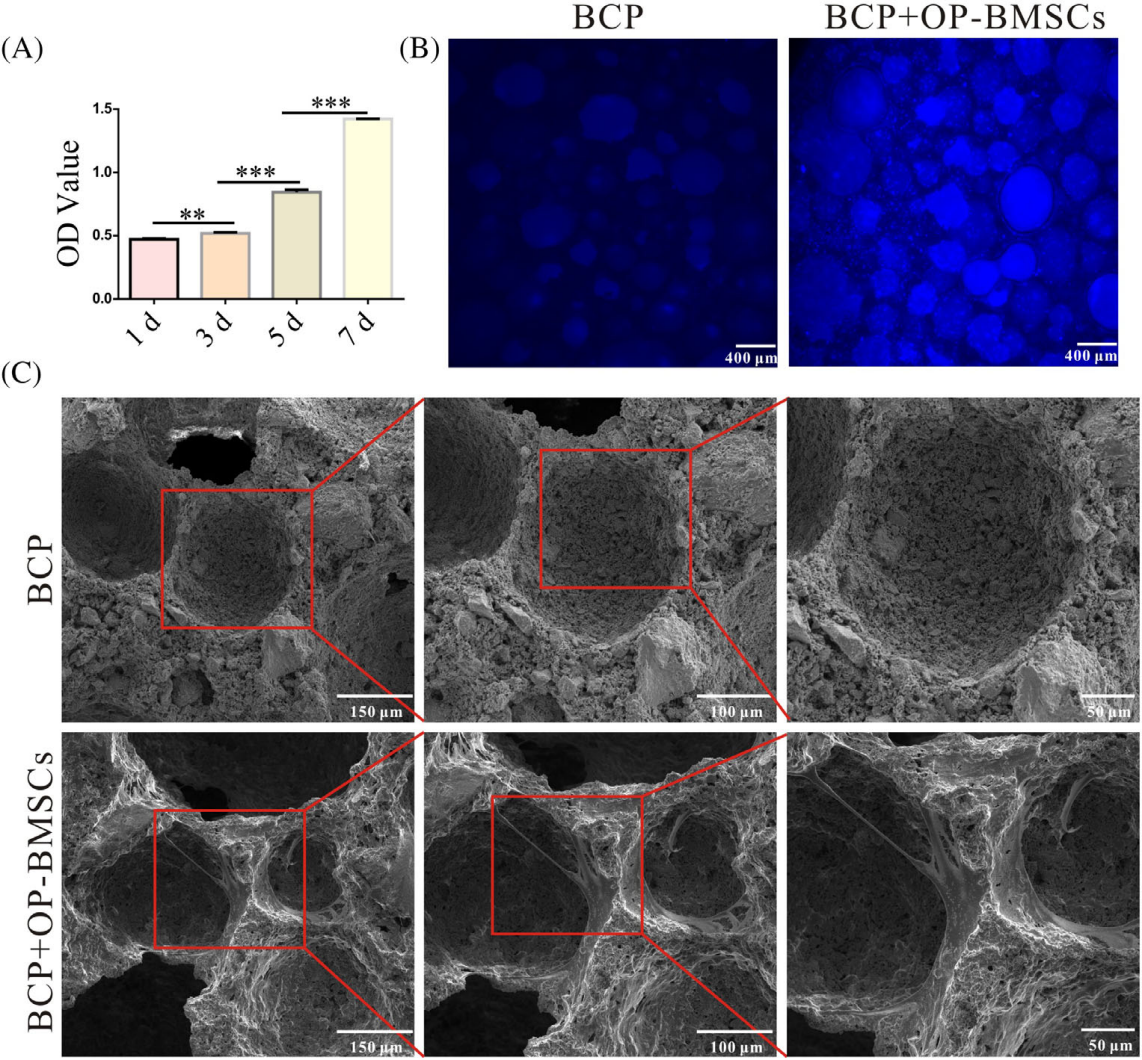
OP‐BMSCs could proliferate and seed on BCP. (A) CCK‐8 detected the proliferation of OP‐BMSCs on BCP at different times. (B) DAPI staining for OP‐BMSCs on BCP. (C) SEM of OP‐BMSCs and BCP for co‐culture of 3 days. Data represent the mean ± SD (*n* ≥ 3). (*p < 0.05, **p < 0.01, ***p < 0.001)

*In vivo*, we implanted BCP with OP‐BMSCs into critical‐sized calvarial defects of OP model rats. First, the CCK‐8 showed that OP‐BMSCs could grow and proliferate on BCP (Figure 6A). Next, we used DAPI staining to confirm that OP‐BMSCs were seeded on BCP. The results showed DAPI‐stained nuclei on BCP seeded with OP‐BMSCs, whilst there was no staining on BCP without OP‐BMSCs (Figure 6B). Furthermore, we used SEM to observe the morphology and growth of OP‐BMSCs on BCP. The results showed that OP‐BMSCs adhered to BCP after co‐culture for 3 days (Figure 6C). Finally, BCP seeded with OP‐BMSCs was transplanted to the critical‐sized calvarial defects of OP rats.

**FIGURE 7:**
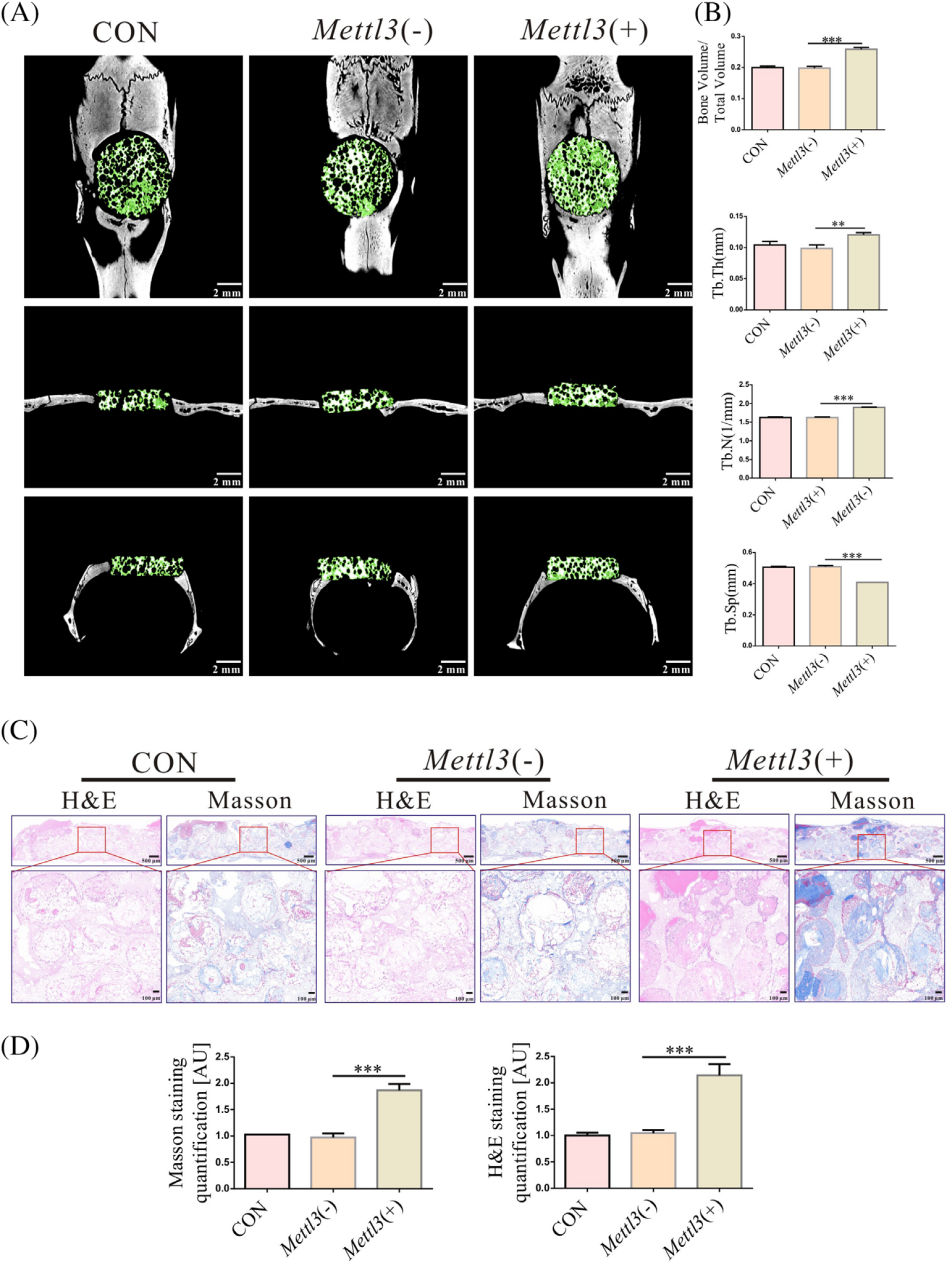
Overexpression of *Mettl3* upregulated the osteogenic ability of OP‐BMSCs *in vivo*. (A) Micro‐CT showed overexpression of *Mettl3* increased the BV/TV, Tb.Th and Tb.N, whilst Tb.Sp was decreased in *Mettl3*(+) group after transplantation of OP‐BMSCs‐seeded BCP for 8 w. (B) H&E and Masson staining showed that the amount of fibrotic and mineralized new bone in *Mettl3*(+) group was more than that in CON, *Mettl3*(−) groups after transplantation of OP‐BMSCs‐seeded BCP for 8 w. (C) Semi‐quantitative analysis of H&E staining and Masson staining by utilizing ImageJ. Data represent the mean ± SD (*n* ≥ 3). (*p < 0.05, **p < 0.01, ***p < 0.001)

Eight weeks after transplantation, micro‐CT results showed significantly increased levels of bone matrix in the *Mettl3*(+) group, the BV/TV, Tb. Th and Tb.N increased in the *Mettl3*(+) group, whilst Tb.Sp was decreased (Figure 7A,B). The new bone matrix was red and more intense staining in the *Mettl3*(+) group than in the *Mettl3*(−) group by H&E. Moreover, the Masson staining detected new bone matrix was blue and more intense in the *Mettl3*(+) group than in the *Mettl3*(−) group results (Figure 7C,D).

Taken together, overexpression of *Mettl3* can activate the Wnt signalling pathway and increase the osteogenic potential of OP‐BMSCs. Thus, *Mettl3* plays an indispensable role and could be an important target for treating OP‐associated bone defects.

## 4 DISCUSSION

OP is a chronic systemic bone disease in postmenopausal women that has become a serious public health problem.^35^, ^36^ The pathological features of OP include increased bone turnover, decreased bone mass, destroyed bone microstructure and decreased bone mineral density (BMD). According to previous reports, hundreds of millions of people worldwide suffer from OP‐related fractures.^13^, ^37^, ^38^ The current methods for treating osteoporotic bone defects include autologous bone transplantation, allogeneic bone transplantation and tissue‐engineered bone treatment. Currently, the role of allogeneic MSCs in various orthopaedic conditions is prospective. A cell bank has been established for regenerative medicine, and cell therapy has become a hot spot in current research.^39^ BMSCs play an important part in bone formation as a precursor of osteoblasts and are the gold standard for MSC tissue engineering treatment.^26^, ^40^, ^41^ However, studies have also shown that the bone formation capability of OP‐BMSCs is more worse than that of BMSCs.^42^, ^43^ We obtained the same results in our experiments. Therefore, it is a worthy research topic to search for important target genes in OP‐BMSCs and promote treatment effect in the bone defect area after OP‐BMSCs implantation.

M^6^A plays important role in the differentiation and development of cell lineages.^44^, ^45^ M^6^A is a bidirectional process that is regulated by methylases and demethylases. METTL3 was identified as an important ingredient of the methyltransferase complex in mammalian cells.^46^ Wu et al. reported that loss of *Mettl3* activity leads to impaired bone formation, and *Mettl3* overexpression in MSCs could protect OP mice.^47^ In this study, the mRNAs of OP‐BMSCs and BMSCs were extracted for dot blot experiments, which revealed decreased methylation levels in OP‐BMSCs compared with those in BMSCs. At the same time, the *Mettl3* was decreased in OP‐BMSCs compared with those in BMSCs. OPN and RUNX2 were also decreased in OP‐BMSCs, as was the osteogenic ability of OP‐BMSCs. Therefore, we hypothesized that the decreased bone differentiation of OP‐BMSCs was related to the downregulation of *Mettl3*. However, the molecular mechanism through which downregulated the bone activity of OP‐BMSCs was related to *Mettl3* knockdown remains unclear and is worthy of our further study and discussion. Wu et al. found that knocking out *Mettl3* reduced Pth1r in MSCs, resulting in a decrease in the overall methylation level of m^6^A and the destruction of parathyroid hormone‐induced osteogenic ability.^47^ Song et al. found that *Metttl3* could activate the MAPK signalling pathway to enhance the osteogenic differentiation of hASCs.^48^ Hong et al. found that *Mettl3* was a bone repair stimulating factor.^49^ Overexpressing *Mettl3* promotes the osteogenic differentiation and migration ability of BMSCs, whilst silencing *Mettl3* directly reduces m^6^A methylation levels, downregulates the expression of osteogenic genes and aggravates OP. However, there are few studies on the mechanism of the osteogenic effects of *Mettl3* in OP‐BMSCs, and further research is needed.

The Wnt signalling pathway is closely related to bone formation and widely known.^50^, ^51^ Our results showed that the expression of β‐catenin, P‐Gsk‐3β and Lef1 was reduced in OP‐BMSCs. Therefore, we speculated that *Mettl3* might upregulate the canonical Wnt signalling pathway and increase bone regeneration of OP‐BMSCs. Thus, we transduced OP‐BMSCs with a *Mettl3* overexpression lentivirus to detect changes of methylation levels, osteogenic factors and Wnt‐related molecules. Our results showed that overexpressing *Mettl3* partially restored the osteogenic ability of OP‐BMSCs, activated the Wnt signalling pathway and increased the expression of Opn, Runx2, P‐Gsk‐3β, β‐catenin and Lef1. Furthermore, this canonical Wnt signalling pathway was inhibited by the canonical Wnt inhibitor DKK1,^52^ which caused reduced mRNA and protein expression of P‐Gsk‐3β, β‐catenin and Lef1 and diminished osteogenic ability. After 3 and 5 days of osteogenic induction, ALP staining showed increased ALP activity after overexpressing *Mettl3*, whilst ALP activity was weakened in the DKK1 group. Alizarin red staining showed a significant increase in mineralization nodules after overexpressing *Mettl3*, and these calcium nodules were weakened by DKK1 treatment. Therefore, *Mettl3* may explain the reason for the decline in the osteogenic ability of OP‐BMSCs and provide a potential therapeutic target for cell therapy to treat OP fractures.

However, the role of *Mettl3* in regulating the osteogenic ability of OP‐BMSCs needs to be further verified *in vivo*. The rat critical‐sized calvarial defect model can be used for *in vivo* experiment.^53^, ^54^, ^55^ A critical‐sized defect is defined as the smallest size wound that does not heal spontaneously within the natural lifespan.^56^ Therefore, we established an OP rat model with a critical‐sized skull defect to study the effect of *Mettl3* on OP‐BMSCs *in vivo*. BMSCs transplantation is a promising method of treating various diseases, including neurological diseases, fractures and many other types of diseases.^57^, ^58^ Yin Tang et al. used the critical size cranial bone defect model implanted with a scaffold seeded with BMSCs, which could enhance local bone formation and be used to treat patients with OP bone defects and fractures.^59^ BCP is porous calcium phosphate ceramic similar to natural bone and has good biocompatibility.^60^, ^61^, ^62^ In this study, we transplanted BCP seeded with OP‐BMSCs into a rat calvarial defect model for *in vivo* validation. Reconstructed micro‐CT and histological results showed that the *Mettl3*(+) group had the greatest amount of new bone at 8 weeks. The results indicated that overexpressing *Mettl3* could rescue the impaired osteogenic ability of OP‐BMSCs. These results were consistent with in vitro experiments.

In summary, the methylation levels and osteogenic potential of OP‐BMSCs were decreased in OP‐BMSCs. Overexpression of *Mettl3* could promote the osteogenic potential of OP‐BMSCs by activating the Wnt signalling pathway. *Mettl3* has the potential to be an important regulatory gene related to the osteogenesis of OP‐BMSCs, providing a new direction for the treatment of bone defects in OP patients.

## CONFLICT OF INTEREST

The authors declare no competing interests.

## AUTHOR CONTRIBUTIONS

Tianli Wu established the OP rat model, performed *in vitro* and *in vivo* experiments, executed the analysis of the data and drafted the manuscript. Hui Tang, Jianghua Yang and Zhihao Yao collected the data. Long Bai and Yuping Xie assisted in the *in vivo* experiments. Qing Li designed the experimental project and revised the manuscript. Jingang Xiao initiated the study, designed the experimental project, analysed data, revised the manuscript and provided funding. All authors have seen and approved the manuscript.

## Data Availability

All data included in this article can be obtained from corresponding author upon reasonable requirements.
